# Complicated Pandemic (H1N1) 2009 during Pregnancy, Taiwan

**DOI:** 10.3201/eid1710.101608

**Published:** 2011-10

**Authors:** Wan-Ting Huang, Yu-Fen Hsu, Tsung-Wen Kuo, Wan-Jen Wu, Jen-Hsiang Chuang

**Affiliations:** Taiwan Centers for Disease Control, Taipei, Taiwan

**Keywords:** pandemic (H1N1) 2009, influenza, viruses, pregnancy, complications, Taiwan, letter

**To the Editor:** Pregnant women with pandemic (H1N1) 2009 virus infection are at increased risk for severe illness and complications (*1**–**3*). Recent reports have shown that this infection causes disproportionate illness and death in pregnant women and has been associated with adverse fetal and neonatal outcomes. We characterized the severity of pandemic (H1N1) 2009 virus infection among pregnant women in Taiwan.

Complicated influenza infection, defined as influenza-like illness and evidence of pneumonia, neurologic symptoms, myopericarditis, or invasive bacterial infections, has been a notifiable disease in Taiwan since 2002 (*4*). We reviewed reports and medical records of complicated pandemic (H1N1) 2009 virus infection, confirmed by real-time reverse transcription PCR in women 15–49 years of age who had onset of illness during July 1–December 31, 2009. Data were obtained for demographics; pregnancy status and outcome; gestational age at illness onset; preexisting medical conditions; onset of illness; treatment; and severity, including intensive care unit (ICU) admission.

To calculate rates of complicated pandemic (H1N1) 2009 virus infection, we estimated the pregnant population during July 1–December 31, 2009, by using the National Health Insurance computerized database for Taiwan (*5*). Women who were 15–49 years of age and had been assigned International Classification of Diseases, 9th Revision, Clinical Modification (www.cdc.gov/nchs/icd/icd9cm.htm), codes of V22* (normal pregnancy) and V23* (supervision of high-risk pregnancy) during the study were considered pregnant. Number of nonpregnant women was estimated by subtracting the calculated number of pregnant women from the number of women 15–49 years of age from 2009 household registration data (*6*). We estimated 95% confidence intervals (CIs) for rates by using exact binomial methods.

During July 1–December 31, 2009, data were reported for 10 pregnant women and 138 nonpregnant women 15–49 years of age who had confirmed, complicated pandemic (H1N1) 2009 virus infections. Dates of illness onset ranged from August 3 through December 31, 2009. Median age of the 10 pregnant women was 24.5 years (range 22–32 years), and median gestational age at illness onset was 24 weeks (range 5–37 weeks). Other than pregnancy, none of these women had high-risk conditions for influenza complications recognized by the Advisory Committee on Immunization Practices (*7*). Seven women gave birth during hospitalization; 4 fetuses were stillborn, and 3 were live-born. At birth, the 3 live-born infants were at 27, 32, and 37 weeks’ gestation and weighed 824, 1,850, and 3,270 g, respectively; all were admitted to a neonatal ICU.

Four (40%) pregnant and 84 (63%) nonpregnant women received oseltamivir treatment within 48 hours of illness onset (p = 0.19) (Table). Acute respiratory distress syndrome developed, mechanical ventilation was required, and extracorporeal membrane oxygenation was required in a higher proportion of pregnant women than nonpregnant women. Median length of hospital stay was 8 days (range 3–47 days) for pregnant women and 5 days (range 0–100 days) for nonpregnant women (p = 0.03). Five (50%) pregnant and 31 (22%) nonpregnant women were admitted to an ICU (p = 0.06); 3 (30%) pregnant women and 5 (4%) nonpregnant women died (p = 0.01).

**Table:** Characteristics of women ages 15–49 y who had confirmed pandemic (H1N1) 2009 infection, by pregnancy status, Taiwan, July 1–December 31, 2009*

Characteristic	Pregnant, n = 10	Nonpregnant, n = 138	p value†
Age, y	24.5 (22–32)	27.5 (15–49)	0.39
ACIP high-risk condition other than pregnancy	0	28 (20)	0.21
Pneumonia	9 (90)	134 (97)	0.30
ARDS	5 (50)	14 (10)	0.003
Admission to hospital	9 (90)	138 (100)	0.07
Time from symptom onset to hospitalization, d	2 (0–7)	2 (1–12)	0.78
Length of hospital stay, d	8 (3–47)	5 (0–100)	0.03
Admission to ICU	5 (50)	31 (22)	0.06
Length of ICU stay, d	16 (6–33)	5 (0–83)	0.07
Oseltamivir treatment	9 (90)	135 (98)	0.25
<48 h after illness onset	4 (40)	84‡ (63)	0.19
Mechanical ventilation	5 (50)	19 (14)	0.01
ECMO	3 (30)	1 (1)	<0.001
Death	3 (30)	5 (4)	0.01
Time from illness onset to death, d	16 (2–37)	9 (1–32)	0.57

*Values are median (range) or no. (%). ACIP, Advisory Committee on Immunization Practices; ARDS, acute respiratory distress syndrome; ICU, intensive care unit; ECMO, extracorporeal membrane oxygenation. †Medians were compared by using Wilcoxon rank-sum test, and proportions were compared by using Fisher exact test. ‡For 4 nonpregnant women, information on date of initiation of oseltamivir treatment was unknown.

There were 168,364 pregnant women and 6,220,197 nonpregnant women 15–49 years of age in Taiwan throughout the study period. The rate of complicated pandemic (H1N1) 2009 virus infection was 5.94 per 100,000 pregnant women (95% CI 2.85–10.92) and 2.22 per 100,000 nonpregnant women (95% CI, 1.86–2.62). Pregnant women were at greater risk for complicated pandemic (H1N1) 2009 virus infection than nonpregnant women (risk ratio 2.68, 95% CI 1.41–5.09).

Findings from this study have several limitations. Ascertainment of patients with complicated pandemic (H1N1) 2009 virus infection relied on passive surveillance. Therefore, data collection varied in completeness and quality between hospitals and different surveillance periods. The small number of pregnant women with confirmed complicated pandemic (H1N1) 2009 virus infection limited statistical power for stratified analyses by patient demographics and other characteristics. On November 1, 2009, Taiwan concurrently began a nationwide vaccination program against pandemic (H1N1) 2009 (*8*). As of December 31, 2009, a total of 8% of pregnant women and 13% of persons >15 years of age had been vaccinated (Taiwan Centers for Disease Control, unpub. data). Calculated rates of complicated pandemic (H1N1) 2009 virus infection could be affected by variable vaccine coverage among pregnant and nonpregnant women.

In Taiwan, oseltamivir treatment was provided free during the 2009 influenza pandemic to patients with influenza-like illness who had positive results for influenza by rapid influenza diagnostic tests, signs that signal progression to severe diseases (*9*), and clinical evidence of complicated influenza infections. The government recommended that pregnant women receive the vaccine against pandemic (H1N1) 2009, regardless of stage of pregnancy, and made this group a priority. Our findings are consistent with those of other studies (*1**–**3*) and suggest that pregnancy is a risk factor for severe or fatal pandemic (H1N1) 2009 virus infection in Taiwan. These findings justify policies to treat and vaccinate pregnant women against pandemic (H1N1) 2009.

## References

[1] Jamieson DJ, Honein MA, Rasmussen SA, Williams JL, Swerdlow DL, Biggerstaff MS, H1N1 2009 influenza virus infection during pregnancy in the USA. Lancet. 2009;374:451–8. 10.1016/S0140-6736(09)61304-0 19643469

[2] Louie JK, Acosta M, Jamieson DJ, Honein MA. Severe 2009 H1N1 influenza in pregnant and postpartum women in California. N Engl J Med. 2010;362:27–35. 10.1056/NEJMoa0910444 20032319

[3] Siston AM, Rasmussen SA, Honein MA, Fry AM, Seib K, Callaghan WM, Pandemic 2009 influenza A(H1N1) virus illness among pregnant women in the United States. JAMA. 2010;303:1517–25. 10.1001/jama.2010.479 20407061 PMC5823273

[4] Chien YS, Su CP, Tsai HT, Huang AS, Lien CE, Hung MN, Predictors and outcomes of respiratory failure among hospitalized pneumonia patients with 2009 H1N1 influenza in Taiwan. J Infect. 2010;60:168–74. 10.1016/j.jinf.2009.12.012 20036689

[5] Bureau of National Health Insurance. National health insurance in Taiwan 2009 [cited 2010 Sep 23]. http://www.nhi.gov.tw/resource/Webdata/Attach_13787_1_NationalHealthInsuranceinTaiwan2009.pdf

[6] Department of Household Registration. End of year statistics, 2009 [cited 2010 Sep 23]. http://www.ris.gov.tw/web_eng/eng_sta.html

[7] National Center for Immunization and Respiratory Diseases, Centers for Disease Control and Prevention (CDC). Use of influenza A (H1N1) 2009 monovalent vaccine: recommendations of the Advisory Committee on Immunization Practices (ACIP), 2009. MMWR Recomm Rep. 2009;58:1–8. 19713882

[8] Huang WT, Chen WW, Yang HW, Chen WC, Chao YN, Huang YW, Design of a robust infrastructure to monitor the safety of the pandemic A(H1N1) 2009 vaccination program in Taiwan. Vaccine. 2010;28:7161–6. 10.1016/j.vaccine.2010.08.069 20804804

[9] World Health Organization. Recommended use of antivirals. Pandemic (H1N1) 2009 briefing note 8 [cited 2010 Sep 23]. http://www.who.int/csr/disease/swineflu/notes/h1n1_use_antivirals_20090820/en/index.html

